# Reference values and physiological characterization of a specific isolated pig kidney perfusion model

**DOI:** 10.1186/1745-6673-2-1

**Published:** 2007-01-29

**Authors:** Volker Unger, Christian Grosse-Siestrup, Claudia Fehrenberg, Axel Fischer, Michael Meissler, David A Groneberg

**Affiliations:** 1 Department of Comparative Medicine and Facilities of Experimental Animal Sciences, Charité – Universitätsmedizin Berlin, Free and Humboldt-University Berlin, Augustenburger Platz 1, D-13353 Berlin, Germany; 2 Allergy-Centre-Charité, Otto-Heubner-Centre, Pneumology and Immunology, Charité – Universitätsmedizin Berlin; Augustenburger Platz 1, D-13353 Berlin, Germany; 3 Institute of Occupational Medicine, Charité – Universitätsmedizin Berlin, Ostpreussendamm 111, D-12207 Berlin, Germany; 4 Department of Respiratory Medicine, Hannover Medical School, Carl-Neuberg-Str. 1 OE 6870, D-30625 Hannover, Germany

## Abstract

**Background:**

Models of isolated and perfused kidneys are used to study the effects of drugs, hazardous or toxic substances on renal functions. Since physiological and morphological parameters of small laboratory animal kidneys are difficult to compare to human renal parameters, porcine kidney perfusion models have been developed to simulate closer conditions to the human situation, but exact values of renal parameters for different collection and perfusion conditions have not been reported so far. If the organs could be used out of regular slaughtering processes animal experiments may be avoided.

**Methods:**

To assess renal perfusion quality, we analyzed different perfusion settings in a standardized model of porcine kidney hemoperfusion with organs collected in the operating theatre (OP: groups A-D) or in a public abattoir (SLA: group E) and compared the data to in vivo measurements in living animals (CON). Experimental groups had defined preservation periods (0, 2 and 24 hrs), one with additional albumin in the perfusate (C) for edema reduction.

**Results:**

Varying perfusion settings resulted in different functional values (mean ± SD): blood flow (RBF [ml/min*100 g]: (A) 339.9 ± 61.1; (C) 244.5 ± 53.5; (D) 92.8 ± 25.8; (E) 153.8 ± 41.5); glomerular fitration (GFR [ml/min*100 g]: (CON) 76.1 ± 6.2; (A) 59.2 ± 13.9; (C) 25.0 ± 10.6; (D) 1.6 ± 1.3; (E) 16.3 ± 8.2); fractional sodium reabsorption (RF_Na _[%] (CON) 99.8 ± 0.1; (A) 82.3 ± 8.1; (C) 86.8 ± 10.3; (D) 38.4 ± 24.5; (E) 88.7 ± 5.8). Additionally the tubular coupling-ratio of Na-reabsorption/O_2_-consumption was determined (T_Na_/O_2_-cons [mmol-Na/mmol- O_2_] (CON) 30.1; (A) 42.0, (C) 80.6; (D) 17.4; (E) 23.8), exhibiting OP and SLA organs with comparable results.

**Conclusion:**

In the present study functional values for isolated kidneys with different perfusion settings were determined to assess organ perfusion quality. It can be summarized that the hemoperfused porcine kidney can serve as a biological model with acceptable approximation to in vivo renal physiology, also if the organs originate from usual slaughtering processes.

## Background

A variety of isolated and perfused kidney models has been used for the study of renal functional parameters [1-6]. If the kidneys are perfused normothermically with autologous blood, they exhibit unique possibilities for pharmacology and toxicology studies and for the improvement of the graft function after transplantation. As the donor kidneys are subject to warm and cold ischemia due to the explantation process and the preservation [7-10], the investigation of ischemia- and reperfusion-related injuries [11-15] which cause a great number of organ failures, is still very important.

While easy in use, the perfusion of small laboratory animal kidneys has often been unsatisfactory since the renal function of these animals largely differ in comparison to the human organ [16-18]. In contrast to the situation in rodent organisms, the functional morphology of porcine kidneys is closer to the situation in humans. Therefore porcine kidney perfusion systems are often used in experimental nephrology [1,19-21].

Next to the renal anatomy and function, a further advantage of porcine organs is based on the availability of organs from commercially slaughtered animals. The use of these slaughterhouse kidneys can lead to the reduction in the number of experimental animals. Legally, slaughterhouse kidney perfusion studies are not defined as animal experiments and therefore fulfill international standards in terms of establishing alternatives to animal experimentations [22].

Many perfusion settings exist for porcine kidney perfusion models but reference values for different perfusion conditions have not been defined so far. Physiological reference values out of in vivo animal studies are of limited meaning for the validation of the isolated kidney function due to the organ's separation from extra-organic nervous and humoral control mechanisms. For example strong poliuric states with urine flow rates of 10 ml/min and more may occur, caused partly by the absence of ADH control in this kidney model.

Therefore the present study was performed, to define comparative values of renal functional parameters in both, laboratory and slaughterhouse harvested isolated porcine kidneys. The organs were studied under different preservation and perfusion conditions and were compared to the in vivo renal function of pigs. Physiologically the focus was set 1.) on the glomerular filtration, determined by the exogenous creatinine clearance [23-25] and 2.) on postglomerular mechanisms, controlling renal sodium handling. Sodium reabsorption is an active, oxygen-consuming process dependent upon sodium potassium pumps [26-28]. This had been studied already for the isolated kidney of the rat [29] and also for the state of postischemic acute renal failure [30]. The metabolic coupling between the sodium reabsorption and the oxygen consumption [31-34] therefore is used here as a further indicator for the performance of the isolated pig kidney.

## Materials and methods

### Animals and experimental groups

After approval of the local official veterinarian institutions, German landrace female pigs (age six months) were used. Six differently treated groups (table 1) were analyzed for reference values. Kidneys from four groups were collected from laboratory animals in an operating theatre (A-D), kidneys of group E originated from slaughterhouse animals at an abattoir. Whereas in group (A) no preservation at all took place, the organs of the groups B-E were preserved before hemoperfusion (B, C, : 2 hrs, D 24 hrs, E about 5 hours due to the process of slaughtering and transport). In group C, albumin was added to the perfusate to approximate physiological colloid osmotic pressure with the two aims: 1.) to normalize effective filtration pressure relations in the glomerula of the kidney and 2.) to reduce the danger of edema.

**Table 1:** Isolated kidney experimental groups

Group	**Organ Harvesting**	**Preservation time (ca. hrs)**	**Preservation medium**	**Oncotic medium**	**Organ numbers**
**A**	OP	no	no	-	16
**B**	OP	2	B2	-	16
**C**	OP	2	B2	ALB	16
**D**	OP	24	B2	-	8
**E**	SLA	5	B2	-	16
**CON**	-	-	-	-	8

(OP: laboratory animal kidneys collected in the operating theatre; SLA: slaughterhouse organs; B2: von Baeyer solution; ALB: albumin added to the perfusate)

The control group (CON) originated from 8 living laboratory animals, kept under controlled conditions for 1 week in the stables of the facility, inhouse with the laboratories and the operation room. The animals were provided with blood access via a cannulated external jugular vein for three days. On the second day of this period the individual animals were hold in a metabolic cage for the purpose of 24 hour urine collection. The individual three day mean values of the blood samples and the 24 hour urine values were used as basic data for the CON group.

Selected results from group CON had already been presented in part in a previously published methodological study [35] to demonstrate a new graphical depiction method.

### Blood collecting

For the collection of blood of the slaughterhouse animals, as previously described in detail [19,36]., the cervical vessels (Venae jugularis dex.et sin., V. cava cranialis) were punctured and the collected blood was anticoagulated with sodium citrate (18 ml/l) and heparine (5.000 IE/l). The blood was then filtered (Biotest TNSB-3 transfusion device, 200 μm) and stored in sterile blood bags [2]. Alternatively, in the laboratory animals group, blood was collected under sterile conditions via the cannulated external jugular vein.

### Organ collecting

The pigs of the slaughterhouse groups were electrically stunned and then exsanguinated. Then the organs were removed by en bloc technique, arterially cannulated and flushed with preservation solution (4°C) containing 5.000 IE/L heparine (Liquemin N, Roche). 500 ml of preservation solution (see table 2 for B2-solution pursuant to von Baeyer [8]) was then applicated into the artery and the kidneys were transferred under sterile, hypothermic (4°C) conditions from the abattoir to the laboratory.

**Table 2:** Preservation solutions [8]

		**B2**	**HTK**	**Euro Collins **
Osmolality (mosm/kg)		323	310	406
				
**Ions **(mmol/l)	Na^+^	22	15	10
	K^+^	150	9	115
	Mg^++^	10	4	
	Cl^-^	10	50	15
	SO4^-^	85		
				
**Buffers **(mmol/l)	PO4^---^	6		
	HPO4^--^			43
	H2PO4^-^			15
	HCO3^-^			10
	Histidin		198	
				
**Osmotics **(mmol/l)	Glucose			198
	Mannitol		30	
	Saccharin	40		
				
**Colloids **(mmol/l)	PEG	25		

Kidneys from laboratory animals were handled in the same way after being removed surgically. For organ harvesting by surgery, pigs were set under general anesthesia undergoing median laparotomy. The right external jugular vein was cannulated and the animal was heparinized (300 IE/kg body weight). Kidneys were removed and cannulated one by one before the animal was exsanguinated. Normally one kidney was perfused immediately and the other underwent the preservation procedure before the reperfusion.

### Perfusion procedures

Perfusion procedures were carried out as previously established for kidneys and other organs [19,37,38]. Ureteral and vascular catheters were implanted and a period of warm rinsing with 500 ml of preservation solution was performed before hemoperfusion with autologous blood was conducted. The hemoperfusion started with an arterial flow of 50–100 ml/min and a mean arterial pressure never allowed to exceed 100 mmHg to ensure an optimal organ warming up and the beginning of renal autoregulation under reperfusion. Blood and urine samples for assessment of parameters were collected after entering a steady state usually after 20–30 min. Then, within clearance periods of 30 min, urine collection and blood sampling was performed and immediately followed by blood gas analysis using an automated blood gas analysator (Radiometer Copenhagen, ABL) to assess pH- and electrolyte status. Further sample fractions were stored for a later transfer to the labotaratory for analysis of multiple other parameters as listed below. Also, venous and arterial pressures and arterial flow were recorded online using ultrasonic flow transducers (Transonic Systems Inc., T206). Organ weight was also assessed directly after surgical resection (prior to eventual cold storage) and before and after reperfusion.

### Perfusion system

The perfusion system consisted of separated blood and dialysis circuits as described [2], that may also be used for the perfusion of other organs and tissues, like the liver [39,40], the heart [41] or the skin [38]. The volume of heparinized (20.000 IE/l) blood was 600 ml, added with standard electrolyte solution (modified Tyrode's solution) to adjust pressures and hemoglobin concentration and to replace urine fluid loss.

The blood was pumped from the reservoir to a low-flux polysulfon dialysis system (model F7, Fresenius, Bad Homburg). Next to dialysis processes, the blood was also oxygenized in this module and then transported to the organ with a second roller-pump. After passage through the organ, the blood reached the reservoir due to hydrostatic pressure differences.

The dialysis circuit containing 10 000 ml of dialysate medium (modified Tyrode's solution) was driven by a roller pump. The dialysate circuit meets the metabolic demands of the organ and, therefore, is permanently oxygenated and nutritional substrates are added as well as creatinine for the determination of the exogenic creatinine-clearance. The substrates are periodically controlled for a steady state in the composition of the dialysate. The temperature was adjusted to 38°C. Controlling of ultrafiltration und thus the perfusate dilution was maintained by continuously weighing the blood reservoir and balancing the afferent and efferent blood roller pumps. The kidneys were kept in a body warm plexi-glass chamber. Urine was collected by way of a ureteral catheter in calibrated glass cylinders.

### Parameters

Apart from basic experimental data (table 3: weight parameters, ischemia time, perfusion time), hemodynamics and blood gases, hemoglobin, blood and urine pH and different electrolytes, the following parameters were measured: free hemoglobin (mg/dl), total blood protein (g/dl), creatinine-concentration in blood (mg/dl) and urine (g/l), urine flow (ml * min^1 ^* 100 g^-1^). By use of the described formulae (see appendix) the following parameters were determined: creatinine clearance (Cl_crea_, ml * min^1 ^* 100 g^-1^), fractional water reabsorption (RF_H2O_, %), fractional sodium reabsorption (RF_Na_, %), tubular sodium transport (T_Na_, mmol * min^-1 ^* 100 g^-1^). Results are presented for the steady state of the model as 60 min values (hematology: table 4; blood, urine laboratory: table 5) and additionally with the 3 hour state for hemodynamics and renal functional parameters (table 6).

**Table 3:** Basic experimental data for isolated kidney experimental groups and for the control group CON of living pigs

group			**CON**	**A**	**B**	**C**	**D**	**E**
**Kidney-Weight**	g	**mean**	**86,1**	**116,9**	**113,1**	**103,9**	**144,1**	**165,3**
		SD	8,3	11,4	17,3	18,6	22,5	57,6
								
**Preservation- Time**	hrs	**mean**	•	•	**2,04**	**2,01**	**24,14**	**5,20**
		SD	•	•	0,35	0,04	1,04	0,84
								
**Warm Ischemia Time**	min	**mean**	•	**7,3**	**8,8**	**6,9**	**7,9**	**17,8**
		SD	•	2,1	2,8	1,6	2,4	7,3
		Signif.)*		**EE**	**E**	**EE**		**D**
								
**Perfusion-Time**	min	**mean**	•	**206,9**	**218,5**	**213,8**	**207,3**	**197,3**
		SD	•	12,9	28,9	10,6	17,3	18,4
								
**Weight Gain Preservation**	%	**mean**	•	•	**10,3**	**11,9**	**5,1**	**9,8**
		SD	•	•	10,2	12,7	5,6	9,4
		Signif.)*			**D**	**D**		**D**
								
**Weight Gain Perfusion**	%	**mean**	•	**39,6**	**28,7**	**15,3**	**26,0**	**31,2**
		SD	•	19,2	17,5	11,1	9,1	21,1
		Signif.)*		**B;CC;D**	**C**	**D**		**D**

(Signif* = Significance is denoted by capital letters labelling the resp. target group for comparison, as well as the level of significance: simple (p < 0.05) = single capital, high (p < 0.01) = twin capitals)

**Table 4:** Hematology values at 60 min hemoperfusion for isolated kidney experimental groups and for the control group CON of living pigs

**Blood**								
		**groups**	**CON**	**A**	**B**	**C**	**D**	**E**

**Hemoglobin**	g/dl	mean	**9,1**	**7,0**	**7,5**	**9,1**	**10,2**	**7,2**
		SD	0,4	1,3	1,4	1,6	2,7	1,5
		Signif.)*	**A;E**					

**Hematocrit**		mean	**0,32**	**0,22**	**0,24**	**0,29**	**0,31**	**0,23**
		SD	0,02	0,04	0,04	0,05	0,09	0,05
		Signif.)*	**A;E**					

**Free Hemoglobin**	mg/dl	mean	**6,1**	**12,9**	**26,8**	**11,4**	**93,0**	**46,8**
		SD	1,3	6,1	33,6	4,1	26,1	29,7
		Signif.)*	**AA**	**DD;EE**	**DD**	**DD;EE**		

**COP**	mmHg	mean	**17,4**	**6,4**	**6,9**	**16,8**	**6,3**	**5,8**
		SD	3,1	1,9	2,9	5,2	2,4	2,2
		Signif.)*	**AA**	**CC**	**CC**	**DD;EE**		

(Signif* = Significance is denoted by capital letters labelling the resp. target group for comparison, as well as the level of significance: simple (p < 0.05) = single capital, high (p < 0.01) = twin capitals)

**Table 5:** Laboratory values for blood and urine at 60 min hemoperfusion of isolated kidney experimental groups and for the control group CON of living pigs

	**Blood**								**Urine**						
	groups		**CON**	**A**	**B**	**C**	**D**	**E**		**CON**	**A**	**B**	**C**	**D**	**E**

**Potassium**	mean	mmol/l	**3,84**	**3,8**	**4,6**	**4,7**	**5,9**	**5,7**	mmol/l	**87,1**	**8,7**	**18,5**	**12,7**	**20,3**	**25,7**
	SD		0,13	0,6	0,4	0,9	0,7	0,9		3,3	6,1	11,9	7,0	17,1	13,9
	Signif.)*		**B;D;E**	**D;E**						**AA;BB;CC; DD;EE**	**B;D;E**				

**Sodium**	mean	mmol/l	**141,7**	**140,7**	**136,2**	**139,1**	**131,2**	**134,7**	mmol/l	**25,1**	**108,9**	**82,2**	**88,8**	**131,1**	**83,9**
	SD		1,2	5,2	4,6	5,2	1,6	3,6		2,5	18,7	16,0	23,6	38,4	24,4
	Signif.)*		**D**	**D**						**AA;BB;CC; DD;EE**	**B;C;D;E**	**D**	**D**		**D**

**Osmolality**	mean	mosm/kg	**291,2**	**281,5**	**283,7**	**288,1**	**275,8**	**289,9**	mosm/kg	**685,9**	**244,8**	**221,4**	**255,2**	**311,5**	**274,7**
	SD		8,4	9,9	7,7	11,4	2,1	8,9		90	24,0	33,1	63,2	133,7	57,6
	Signif.)*									**AA;BB;CC; DD;EE**	**D**	**D**	**D**		

**Creatinin**	mean	mg/dl	**1,05**	**2,5**	**3,4**	**3,5**	**4,9**	**3,7**	g/l	**0,98**	**0,13**	**0,15**	**0,34**	**0,08**	**0,22**
	SD		0,12	0,7	0,6	0,4	1,5	0,9		0,13	0,07	0,07	0,41	0,06	0,08
	Signif.)*		**A;B;C;D;E**	**D**						**AA;BB;C; DD;EE**	**C**	**C**	**D**		**D**

**Urea**	mean	mg/dl	**21,1**	**19,1**	**22,6**	**22,7**	**27,8**	**24,5**	g/l	**17,6**	**0,66**	**0,74**	**1,13**	**0,58**	**0,96**
	SD		1,5	3,9	2,5	1,7	3,0	2,2		4,0	0,18	0,28	0,38	0,34	0,17
	Signif.)*									**AA;BB;CC; DD;EE**	**C**		**D**		**D**

**Glucose**	mean	mg/dl	**109,4**	**135,3**	**115,8**	**124,6**	**112,8**	**112,3**	g/l	**<= 0,1**	**0,24**	**0,17**	**0,38**	**1,13**	**0,63**
	SD		9,2	17,9	15,0	22,7	9,5	6,7			0,19	0,14	0,29	0,16	0,34
	Signif.)*									**(AA;BB;CC; DD;EE)**	**DD;E**	**DD;E**	**DD**		**D**

**Protein**	mean	g/dl	**5,4**	**3,8**	**4,0**	**5,7**	**3,8**	**2,7**	mg/l	**117**	**233**	**435**	**1087**	**10008**	**1862**
	SD		0,2	0,9	1,0	1,0	0,8	0,8		25	207	423	1203	3570	2340
	Signif.)*		**A**	**C**				**C**		**A;BB;CC; DD;EE**	**B;C;DD;E**	**C;DD;E**	**D**		**D**

**pH**	mean		**7,38**	**7,44**	**7,39**	**7,55**	**7,53**	**7,53**		**6,13**	**6,89**	**6,70**	**7,07**	**6,31**	**6,88**
	SD		0,15	0,17	0,09	0,24	0,08	0,09		0,19	0,24	0,33	0,26	0,47	0,39
	Signif.)*									**A;B;C;E**					

(Signif* = Significance is denoted by capital letters labelling the resp. target group for comparison, as well as the level of significance: simple (p < 0.05) = single capital, high (p < 0.01) = twin capitals)

**Table 6:** Hemodynamic and renal functional parameters at 60 and 180 min hemoperfusion of isolated kidney experimental groups and for the control group CON of living pigs

		group	**CON**	**A**	**B**	**C**	**D**	**E**
**RBF **Bloodflow	ml/min*100 g	60 min	●	**339.9**	**224.8**	**244.5**	**92.8**	**153.8**
		SD	•	61.1	28.4	53.5	25.8	41.5
		Signif.)*		**BB;CC;DD;EE**	**DD;E**	**DD;EE**		**D**
		180 min	●	**363.0**	**241.1**	**285.5**	**107.9**	**160.1**
		SD	•	58.0	19.4	48.5	28.4	54.8

**R **Organ- Resistance	mmHg/(ml/min*100 g)	60 min	●	**0.29**	**0.44**	**0.37**	**1.26**	**0.61**
		SD	•	0.05	0.06	0.11	0.49	0.17
		Signif.)*		**BB;C;DD;EE**	**DD;E**	**DD;EE**		**DD**
		180 min	●	**0.28**	**0.4**	**0.29**	**1.01**	**0.61**
		SD	•	0.08	0.03	0.07	0.31	0.19

**O2-cons **Oxygen- Consumption	μmol/min*100 g	60 min	●	**263.9**	**214.3**	**141.6**	**120.8**	**206.4**
		SD	•	49.4	22.3	21.6	27.6	43.5
		Signif.)*		**BB;CC;DD;EE**	**CC;DD**	**EE**		**DD**
		180 min	●	**246.4**	**213.6**	**142.9**	**116.2**	**198.9**
		SD	•	39.4	22.9	19.0	27.6	36.8

**VU **Diuresis	ml/min*100 g	60 min	**0.87**	**13.4**	**7.2**	**5.1**	**0.7**	**3.0**
		SD	0.25	6.1	4.7	3.8	0.3	2.3
		Signif.)*	**AA**	**BB;CC;DD;EE**	**DD;E**	**DD;E**		**DD**
		180 min	●	**14.4**	**8.9**	**5.7**	**0.4**	**3.2**
		SD	•	6.2	5.1	3.2	0.3	2.4

**Cl**_**crea **_Creatinine Clearance	ml/min*100 g	60 min	**76.1**	**59.2**	**27.6**	**25.0**	**1.64**	**16.3**
		SD	6.2	13.9	7.5	10.6	1.26	8.2
		Signif.)*	**A**	**BB;CC;DD;EE**	**DD;EE**	**DD;E**		**DD**
		180 min	●	**65.9**	**30.5**	**24.1**	**1.04**	**15.2**
		SD	•	10.5	4.8	7.5	0.89	9.7

**FF **Filtration- Fraction	%	60 min	●	**22.5**	**15.7**	**14.9**	**2.7**	**13.3**
		SD	•	7.2	6.7	5.3	2.5	6.4
		Signif.)*	•	**B;CC;DD;EE**	**DD**	**DD**		**DD**
		180 min	●	**24.8**	**16.4**	**11.7**	**1.1**	**11.9**
		SD	•	7.2	2.5	3.6	1.1	7.2

**RF**_**H2O **_Water- Reabsorption- fraction	%	60 min	**98.9**	**76.7**	**72.4**	**79.6**	**35.4**	**81.6**
		SD	0.3	9.5	12.8	13.2	31.3	17.2
		Signif.)*	**AA**	**DD**	**DD;E**	**DD**		**DD**
		180 min	●	**76.0**	**70.9**	**72.6**	**36.1**	**74.4**
		SD	•	11.1	15.8	23.3	31.6	30.0

**RF**_**Na **_Sodium- Reabsorption- fraction	%	60 min	**99,8**	**82,3**	**83,1**	**86,8**	**38,4**	**88,7**
		SD	0,1	8,1	10,4	10,3	24,5	5,8
		Signif.)*	**AA**	**DD**	**DD**	**DD**		**DD**
		180 min	●	**80,9**	**79,4**	**81,0**	**46,5**	**89,4**
		SD	•	7,9	13,9	18,3	31,4	6,0

**T**_Na _Sodium- Reabsorption	mmol/min*100 g	60 min	**10,8**	**6,83**	**3,16**	**2,91**	**0,12**	**1,98**
		SD	1,0	2,1	1,0	1,3	0,1	1,08
		Signif.)*	**AA**	**BB;CC;DD;EE**	**DD**	**DD**		**DD**
		180 min	●	**7,82**	**3,43**	**2,77**	**0,09**	**2,02**
		SD	•	1,6	0,7	1,0	0,1	1,1

(Signif* = Significance is denoted by capital letters labelling the resp. target group for comparison, as well as the level of significance: simple (p < 0.05) = single capital, high (p < 0.01) = twin capitals)

### Constructing the diagram (figure 1)

**Figure 1:**
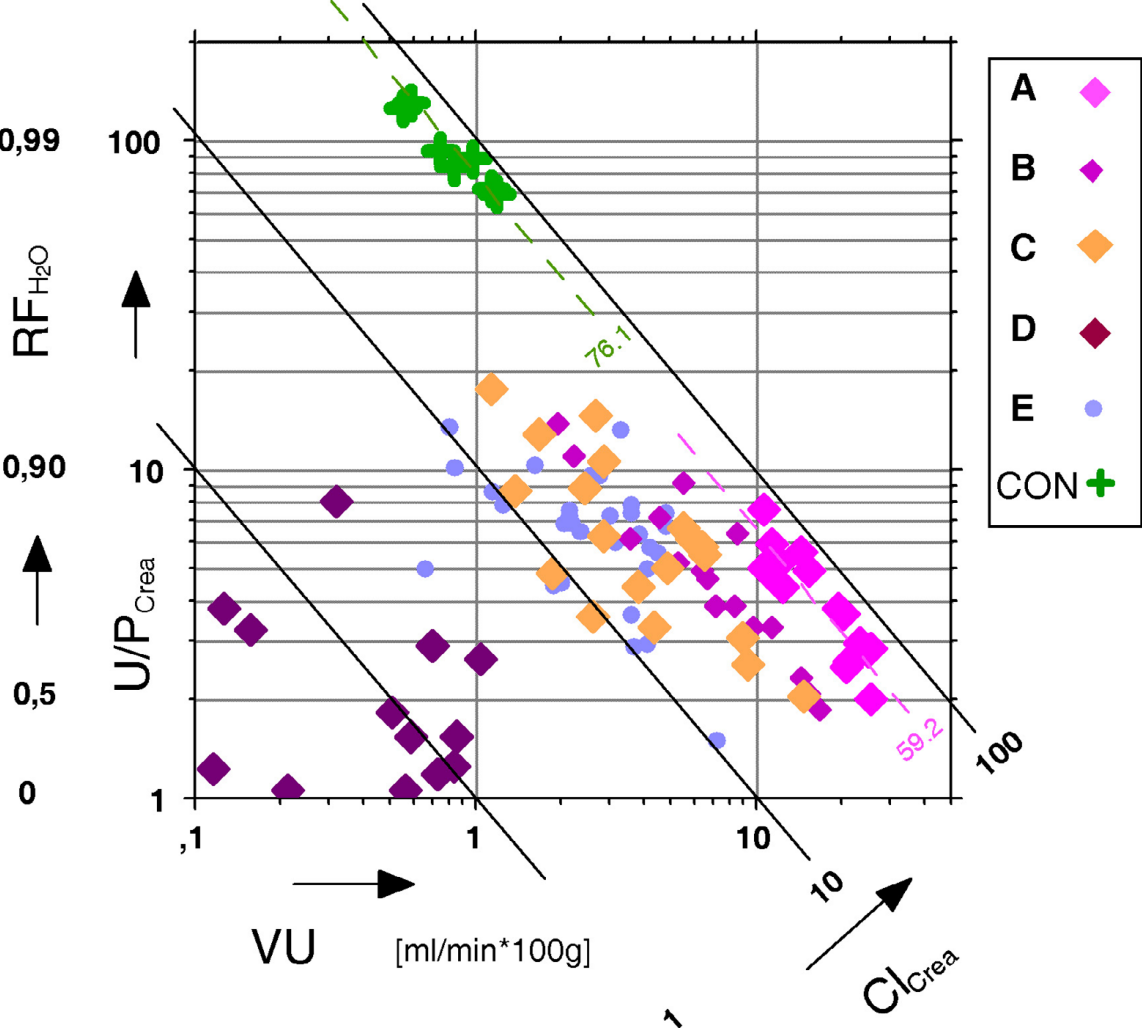
The U/P quotient of creatinine **U/P**_**crea **_versus urine flow **VU **for isolated kidney experimental groups A – E and for the control group of living pigs (CON) (**Cl**_**crea **_= clearance of creatinine; **RF**_**H2O **_= fractional water reabsorption).

To analyze the influence of multiple determands on complex kidney function parameters, a grapho-analytical method was used, which is described in detail in a previously published article for analyzing nephrological parameters [35]. This nomogram-like method is applied here to examine the creatinine clearance used as approximation of the glomerular filtration rate (GFR).

The creatinine clearance represents the mathematical product of the U/P_crea _quotient and the urine-flow VU. Directly displaying these two terms in a x-y diagram leads to certain curves for similar Cl_crea_. values in each experimental group, which are difficult to be distinguished from each other. Therefore the x, y data are transformed into logarithmic scaling and linear lines instead of curves are resulting for constant values of the creatinine clearance. In that way figure 1 was constructed and the interrelation of the following parameters can be analyzed: creatinine U/P quotient (U/P_crea_), urine-flow (VU), creatinine-clearance (Cl_crea_). As a fourth parameter, the fractional reabsorption of water RF_H2O_(see appendix for the formula) can be displayed, since the reciprocal expression of the U/P_crea _quotient, arranged as (1- P/U_crea_), represents the water reabsorption along the tubular system which is numerically present in the second scale of the y-axis in figure 1.

### Statistics

All assessed data are expressed as mean ± standard deviation (SD). Statistical significance (p < 0.05) was tested using StatView 4.5 for Apple Macintosh: the Mann-Whitney-U test for interindividual (group) differences, the Wilcoxon matched pairs test for intraindividual (pairwise) testing and ANOVA regression statistics.

## Results

Value differences determined as statistically significant (p < 0.05) are denoted in the tables and notation is explained in the respective captions in detail.

### General parameters

The basic experimental data are presented in table 3. The studies in groups A-D with kidneys obtained in the operating theatre (OP) were performed under comparable conditions regarding animals, organs, harvesting protocols and warm ischemia time. The latter is significantly increased in group E, the abattoir originating organs (SLA) (table 3). The weight gain of the organs after preservation shows a homogenous range of about 10 % with the significant exception of group D (5.1 %). The weight gain of the organs after reperfusion exhibits comparable values of about 30 % for groups B, D, E. Significant alterations were found for group A with 39.6 % and with a decrease to 15.3 % for the albumin group C.

### Blood and urine parameters

Hematology values are presented in table 4. The hemoglobin (and also the hematocrit in direct proportionality) shows comparable value levels of about 7 g/dl for groups A, B, E, increased values of 9.1 g/l for groups CON, C and a maximum of 10.2 g/l for group E. The free plasma hemoglobin exhibits the lowest value of 6.1 mg/dl in the CON-group, light elevated values of 11.4 mg/dl (group C) and 12.9 mg/dl (group A) and significant alterations from this level for groups E (46.8 mg/dl) and D (93 mg/dl). The colloid osmotic pressure (COP) shows a comparable value level of around 6 mmHg for groups A, B, D, E with significant exceptions for group CON (17.4 mmHg) and the albumin group C (16.8 mmHg).

Laboratory parameters for both blood and urine are presented in table 5 for the collection time at 60 min after start of the perfusion. Generally the blood parameters were kept in approximation to the physiological ranges by periodically controlling the composition of the dialysate (see methods section) and therefore no significant alterations were found, with the exception of creatinine. Creatinine was added to the perfusate for the purpose of determination of the exogenous creatinine clearance, resulting in 3–4 fold concentration levels in comparison to the natural blood values, determined as 1.05 mg/dl in the CON-group.

For all measured urine parameters the situation between the control group CON and all experimental groups A-E is characterized by statistically strong significant (p < 0.01) differences (compare table 5). Additionally there were some significant value differences between single experimental groups for the following parameters:

Potassium concentration with 8.7 mmol/l for group A was found significantly lower than the values for groups B (18.5 mmol/l), D (20.3 mmol/l) and E (25.7 mmol/l). Sodium for group A (108.9 mmol/l) was significantly different from lower values in groups B, C, E and also from the increased value measured for group D (131.1 mmol/l). Creatinine concentration ranged between 0.13 and 0.15 g/l for groups A and B and differed significantly from this level in group C (0.34 g/l) and D (0.08 g/l).

Urea showed a value range from 0.58 to 0.74 g/l for groups A, B, D with a significant difference for group C (1.13 g/l).

A glucose concentration range between 0.17 and 0.38 g/l for groups A, B, C was significantly surpassed in group D (1.13 g/l).

Protein urine concentration measurements revealed three groups with significantly increased levels: group C (1.09 g/l), D (10.0 g/l) and E (1.86 g/l) when compared to groups A and B with a value range from 0.23 to 0.44 g/l.

### Functional parameters

Table 6 shows functional parameters for the hemodynamics, oxygen consumption and for the renal functions at two perfusion time levels: 60 and 180 min. Value differences determined as statistically significant are denoted in table 6 in detail.

#### Hemodynamics

Hemodynamics were kept in controlled constant ranges along the group internal perfusion course concerning the arterial blood pressure, never allowed to exceed 100 mmHg in the mean. Large intergroup differences in the organ vascular resistances R are therefore reflected in significant differences of the blood flow with a maximum value at group A (339.9 ml/min*100 g) and a minimum at D (92.8 ml/min*100 g). A decreasing vascular resistance in all experimental groups during the perfusion course allowed the blood flow to increase within 5–17 % (maximal in group C) between the 60 min and the 180 min state.

#### Oxygen consumption (O_2 _cons)

The oxygen consumption exhibits analogy to the described hemodynamic situation at the 60 min state with values ranging between 263.9 μmol/min*100 g (group A) and 120.8 μmol/min*100 g (D).

*Hemodynamics and oxygen consumption were not measured in the control animals (CON)*.

#### Diuresis (VU)

The diuresis was 15-fold in group A compared to the control value of intact animals (0.9 ml/min*100 g). The other groups ranged between 3.0 (group E) and 7.2 ml/min*100 g (group B). In group D a minimum of 0.7 ml/min*100 g was measured.

#### Creatinine clearance (Cl_crea_)

Creatinine clearance values reached approx. 80% of the control (76.1 ml/min*100 g) in group A (59.2 at 60 min, 65.2 ml/min*100 g at 180 min) and dropped to 2% in group D.

#### Water reabsorption fraction (RF_H2O_)

The fractional reabsorption of water showed levels between 70–80 % of the control in groups A-C, E and a minimum of 35% in group D.

#### Sodium reabsorption fraction (RF_Na_)

The sodium fractional reabsorption for all groups was found to be nearer to the control level then that of water: with maximal values in groups E (88.7 %) and group C (86.8 %) and a minimum at group D (38.4 %).

#### Sodium transport (T_Na_)

The absolute sodium reabsorption paralleled the creatinine-clearance value courses with 10.8 mmol/min*100 g for the control group (CON) and with values between 6.8 (group A) and 0.12 mmol/min*100 g for group D.

## Discussion

Standards in kidney transplantation have been significantly improved during the past years [7,42-44]. They were accompanied by a large number of experimental studies using animal kidney perfusion models [1-5,16,45,46] However, exact reference values for different perfusion conditions have not been described so far and the present studies aimed to address this issue by defining reference values of renal functional parameters in both laboratory and slaughterhouse animal kidneys under different perfusion conditions.

When analyzing the blood parameters of the perfusion groups, a slight increase for free plasma hemoglobin was found in all groups. This increase can be explained by a moderate cell damage by the blood pumps which is commonly found in perfusion systems [4,41]. Also, there was a slight decrease in total blood protein in all perfusion groups that might be explained by protein adsorption at the perfusion system tubes [47] and a certain urinary protein excretion. Likewise, the slight decrease in blood hemoglobin can be explained by a loss of erythrocytes due to blood sampling as previously found in different perfusion settings [19,41].

Kidney function was studied at first at the level of glomerular filtration and four parameters: creatinine U/P quotient (U/P_crea_), urine-flow (VU), creatinine-clearance (Cl_crea_) and water-reabsorption (RF _H2O_) were analyzed by help of a special grapho-analytical method (figure 1).

This separating analysis is crucial since the Cl_crea _is commonly used as the approximation of the glomerular filtration rate and thus can be taken as one of the principal indicators of renal function quality with a physiological mean value in the control group of 76.1 ml/min*100 g (table 6), represented in figure 1 as the dotted green line and as cross symbols for the single measurements. In comparison to this physiological in vivo control, the measurements for group A kidneys, presented in figure 1 and table 6, resulted in a mean value of 59.2 at 60 min of perfusion duration what is fairly comparable to the control level of the creatinine clearance.

Comparable levels of Cl_crea_, as depicted in figure 1, means that the different values arrange along straight declining lines in the nomogram. Using this approach, two hypergroups or clusters of kidneys were found (figure 1): The first cluster containing groups CON and A arrange in a falling linear band (dotted lines) between 60–80 ml/min*100 g. The second cluster consists of groups (B, C, E) showing a broader Cl_crea _value scattering than CON and A with a range of mean values between 27.6 ml/min*100 g (B) and 16.3 ml/min*100 g (E). A minimum Cl_crea _of 1.6 ml/min*100 g was found in group D.

Focussing only on the parameter creatinine-clearance, group A seems to contain the best performing experimental kidneys so far. This could be supposed since group A consists of OP kidneys with no preservation at all.

Comparing the closely related Cl_crea _levels of groups CON and A, however, one has to consider the different underlying physiological conditions (see figure 1): In the CON group the Cl_crea _values are determined under a normal diuresis of about 1 ml/min*100 g with an U/P_crea _of about 100. In group A, contrarily a strong poliuric state is present under isolated perfusion conditions with a 15-fold increased diuresis. Concomitant with this finding an extremely low U/P_crea _value of 5.2 indicates a significantly reduced water reabsorption RF _H2O _of 76.7 % (against 98.9 % in the control kidneys). This can be explained in part by the absence of ADH control in the isolated kidney [5,48].

After kidney function analysis on the basis of these 4 parameters it seems to be doubtful to define group A as best performing in the sense of comparability to normal organ function in living animals. Rather one could presume that isolated organs partly follow their own rules, thus exhibiting a functional behaviour what could be defined as "free-running". This state is due to the kidney's secession from any higher organ control (humoral and nervous system).

With regard to the oppositional postglomerular water flow situations, observed between group A and the control group, it seems to be necessary, to consider further renal parameters and also functional and metabolic mechanisms to qualify the outcome of the isolated kidney.

In this respect, considering the nephron's handling of substrates, the tubular reabsorption of sodium is a further prominent mechanism and the fractional reabsorption of sodium RF_Na _is the representative parameter for this function.

An application of RF_Na _as an useful indicator to qualify renal function under isolated organ perfusion has been demonstrated in a previously published study [35] for isolated pig kidneys with differing experimental protocols.

In the experiments of the actual study however, the RF_Na _value means of groups A, B, C, and E exhibit almost equal levels in a range from 82.3 % (A) to 88.7 % (E) without any significant differences. Only group D separates significantly with a low value of 38.4 %.

**Figure 2:**
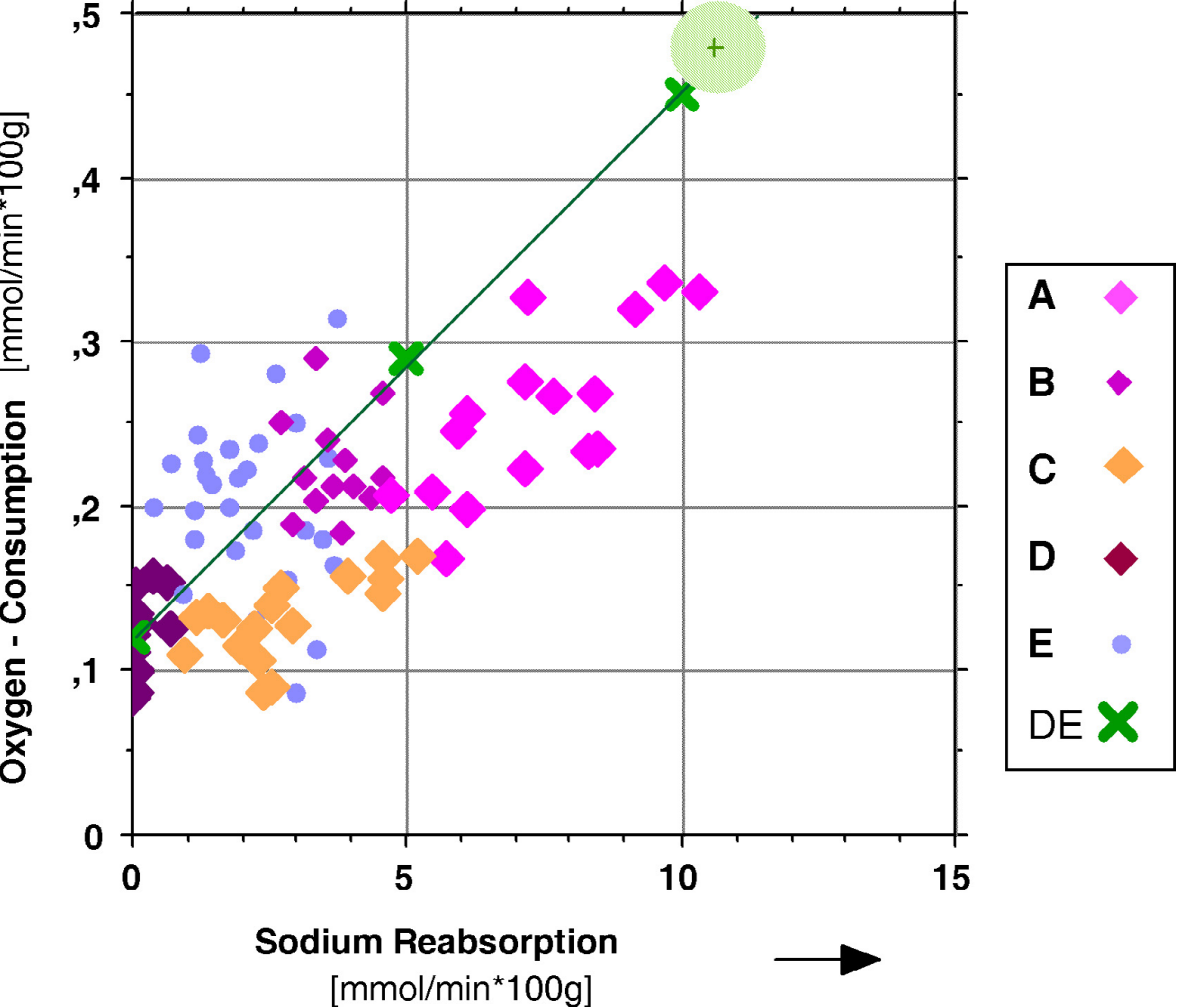
Oxygen consumption **O**_**2 **_**cons **versus fractional sodium reabsorption **RF**_**Na **_for isolated kidney experimental groups A – E, for the control group CON of living pigs (green shadowed area) and for in vivo measurements DE (modified from: [50]).

Sodium reabsorption is an energy consuming process and the physiological coupling between sodium reabsorption and oxygen consumption appeared to be a further promising tool to analyze renal sodium handling in the isolated kidney. This process is described in the literature as of linear proportionality for the mammalian kidney [31-34,49]. That relation is illustrated in figure 2 and table 7 for the experimental groups of kidneys examined in this study : The line which connects the cross symbols (denoted DE) in figure 2 in an almost ideal regression between the oxygen consumption on the y-axis and the sodium reabsorption on the x-axis, represents the physiological in-vivo situation. That part of the diagram (group DE) was adapted from in vivo studies [50] resulting in the following regression equation:

**Table 7:** Regression equations and T_Na_/O_2 _cons qotient of isolated hemoperfused porcine kidneys and of kidneys in alive animals (DE)

group	equation of regression (O_2_cons = a + b * T_Na_)	R^2^	quotient T_Na_/O_2 _cons
**A**	= 0.081 + 0.024 * T_Na_	0.56	42.0
**B**	= 0.131 + 0.02 * T_Na_	0.06	50.6
**C**	= 0.098 + 0.012 * T_Na_	0.43	80.6
**D**	= 0.109 + 0.058 * T_Na_	0.16	17.4
**E**	= 0.138 + 0.042 * T_Na_	0.05	23.8
**DE**	= 0.121 + 0.033 * T_Na_	0.97	30.1

**O**_**2**_-consumption = 0.121 + 0.0332 * T_**Na**_.

The first term on the right side of the equation (0.121) represents the basal oxygen consumption of the kidney without any sodium transport at all. The second term in its reciprocal expression equals in the following value: 30.1 mmol Na/mmol O_2_, representing the number of Na-ions per oxygen-molecule actively and O_2 _-consuming being transported back into the blood. The equation and the values are very similarly reported in other studies [32,33,51].

Out of the isolated kidney groups in figure 2 there were found statistically acceptable (R^2 ^> 0.4) regression lines only for groups A and C (see table 7) with the following T_Na_/O_2 _cons qotients: group A 42.0 and group C: 80.6.

Taking the slope of the green line in figure 2 as the in vivo standard, steeper angles of regression lines, as could be constructed for groups D, E, would result in values of the T_Na_/O_2 _cons coupling qotient lower than the normal 30.1 mmol Na/mmol O_2_. This situation is sometimes discussed as "decoupled". Because there is more than normal oxygen per unit sodium consumed, the generation of heat shock proteins (HSPs) is proposed [52] as one reason of that imbalance. These HSPs play a major roll in renal ischemia and reperfusion injury [14,53], as occurring in perfusion studies, here for the experimental groups D (longest cold ischemia) and E (longest warm ischemia).

In contrast to this situation, as found for groups A, B, C, reduced slopes of regression lines (resulting in higher T_Na_/O_2 _cons quotients, with more than the physiologically normal 30.1 Na-ions per consumed O_2_-molecule, appearing in the renal venous blood), may represent tubular leakage processes [30,54].

## Conclusion

The isolated perfused porcine kidney model used in our experiments, displays a useful approach towards simulating renal functions, even if the organs are collected at a commercial abattoir. It was the aim of the present study to assess renal perfusion quality under specific settings. The perfusion is affected by numerous influences and as presently indicated, large differences in renal function may appear. To evaluate the functional performance of isolated perfused kidneys, besides classical clinical parameters such as the glomerular filtration rate, water and sodium excretion, one additionally should use metabolic efficiency indices as presently discussed. While the model offers a simple way for studying whole organ functional alterations after interventions of clinical or experimental interest, caution should be paid to the exact interpretation of data.

## Abbreviations

**Table 8:** 

**Renal blood flow **	RBF
Renal plasma flow (Hct = hematocrit)	RPF = RBF * (1-Hct)
**Renal resistance**	R = (p_arterial _– p_venous_)/RBF
**Renal oxygen consumption **	(O_2_cons)
Hemoglobin-bound O_2_cons	*O*_2_cons_*chem *_= * RBF *× *Hb *× 1.34 × (*SO*_2*a *_- *SO*_2*v*_)
Physical (soluted) O_2_cons	*O*_2_*cons*_*phys *_= (RPF_*a *_× *pO*_2*a *_- RPF_*v*_× *pO*_2*v*_) × 0.024/760
Total O_2_cons	*O*_2_*cons*_*total *_= *O*_2_*cons*_*chem *_+ *O*_2_*cons*_*phys*_
**Filtration**	
Glomerular filtration rate	GFR ≈ Cl_crea _= U/P_crea _* VU
Filtration fraction	FF = GFR/RPF
Load of substance x	L_x _= GFR * P_x_
**Tubular reabsorption/secretion**	
Transport (absolute)	T_x _= L_x _- E_x_
Fractional reabsorption (relative)	RF_x _= T_x_/L_x_
Reabsorption fraction for water	RFH2O=(1−(1UPcrea)) MathType@MTEF@5@5@+=feaafiart1ev1aaatCvAUfKttLearuWrP9MDH5MBPbIqV92AaeXatLxBI9gBaebbnrfifHhDYfgasaacH8akY=wiFfYdH8Gipec8Eeeu0xXdbba9frFj0=OqFfea0dXdd9vqai=hGuQ8kuc9pgc9s8qqaq=dirpe0xb9q8qiLsFr0=vr0=vr0dc8meaabaqaciaacaGaaeqabaqabeGadaaakeaacqqGsbGucqqGgbGrdaWgaaWcbaGaeeisaG0aaSbaaWqaaiabikdaYaqabaWccqqGpbWtaeqaaOGaeyypa0ZaaeWaaeaacqaIXaqmcqGHsisldaqadaqaamaaliaabaGaeGymaedabaWaaSaaaeaacqqGvbqvaeaacqqGqbauaaGaee4yamMaeeOCaiNaeeyzauMaeeyyaegaaaGaayjkaiaawMcaaaGaayjkaiaawMcaaaaa@4137@
Reabsorption fraction for sodium	RFNa=(1−(UPNaUPcrea)) MathType@MTEF@5@5@+=feaafiart1ev1aaatCvAUfKttLearuWrP9MDH5MBPbIqV92AaeXatLxBI9gBaebbnrfifHhDYfgasaacH8akY=wiFfYdH8Gipec8Eeeu0xXdbba9frFj0=OqFfea0dXdd9vqai=hGuQ8kuc9pgc9s8qqaq=dirpe0xb9q8qiLsFr0=vr0=vr0dc8meaabaqaciaacaGaaeqabaqabeGadaaakeaacqqGsbGucqqGgbGrdaWgaaWcbaGaeeOta4Kaeeyyaegabeaakiabg2da9maabmaabaGaeGymaeJaeyOeI0YaaeWaaeaadaWccaqaamaalaaabaGaeeyvaufabaGaeeiuaafaaiabb6eaojabbggaHbqaamaalaaabaGaeeyvaufabaGaeeiuaafaaiabbogaJjabbkhaYjabbwgaLjabbggaHbaaaiaawIcacaGLPaaaaiaawIcacaGLPaaaaaa@4421@
**Excretion**	
Excretion of water = urine flow	VU
Excretion of substance x	E_x _= VU * U_x_
Quotient U/P for substance x	U_x_/P_x_
(concentration of substance x:	
P_x_- plasma ; U_x_- urine)	

## Conflict of interest statement

The author(s) declare that they have no competing interests.
